# High glycemic load diet, milk and ice cream consumption are related to acne vulgaris in Malaysian young adults: a case control study

**DOI:** 10.1186/1471-5945-12-13

**Published:** 2012-08-16

**Authors:** Noor Hasnani Ismail, Zahara Abdul Manaf, Noor Zalmy Azizan

**Affiliations:** 1 Dietetic Program, School of Healthcare Sciences, Faculty of Health Sciences, Universiti Kebangsaan Malaysia, Jalan Raja Muda Abdul Aziz, 50300, Kuala Lumpur, Malaysia; 2 Department of Dermatology, Hospital Kuala Lumpur, 50300, Kuala Lumpur, Malaysia

**Keywords:** Acne vulgaris, Diet, Glycemic index, Young adults, Dairy products

## Abstract

**Background:**

The role of dietary factors in the pathophysiology of acne vulgaris is highly controversial. Hence, the aim of this study was to determine the association between dietary factors and acne vulgaris among Malaysian young adults.

**Methods:**

A case–control study was conducted among 44 acne vulgaris patients and 44 controls aged 18 to 30 years from October 2010 to January 2011. Comprehensive acne severity scale (CASS) was used to determine acne severity. A questionnaire comprising items enquiring into the respondent’s family history and dietary patterns was distributed. Subjects were asked to record their food intake on two weekdays and one day on a weekend in a three day food diary. Anthropometric measurements including body weight, height and body fat percentage were taken. Acne severity was assessed by a dermatologist.

**Results:**

Cases had a significantly higher dietary glycemic load (175 ± 35) compared to controls (122 ± 28) (p < 0.001). The frequency of milk (p < 0.01) and ice-cream (p < 0.01) consumptions was significantly higher in cases compared to controls. Females in the case group had a higher daily energy intake compared to their counterparts in the control group, 1812 ± 331 and 1590 ± 148 kcal respectively (p < 0.05). No significant difference was found in other nutrient intakes, Body Mass Index, and body fat percentage between case and control groups (p > 0.05).

**Conclusions:**

Glycemic load diet and frequencies of milk and ice cream intake were positively associated with acne vulgaris.

## Background

Acne vulgaris affects up to 85% of adolescent population in the United Kingdom [1]. In Malaysia, the prevalence of facial acne vulgaris among teenagers was 67.5% [2]. The condition was more common among males (71.1%) compared to females (64.6%). Previous studies enquiring into the potential link between diet and acne vulgaris have shown controversial results. Historically, milk was found to be positively associated with acne flares [3] while in 1969, a study reported that there was no association between chocolate and acne vulgaris [4]. However, the crossover study by Fulton et al. [4] was methodologically flawed by comparing chocolate and sweet vegetable oil bars that had the same glycemic index (GI). In addition, the placebo bars had higher content of partially hydrogenated vegetable fat, which may contribute to inflammation due to the trans-fatty acids [5]. This is because, in prostaglandins production, competition between trans and essential fatty acids may lead to inflammation [6]. Later, in the early 70’s, a cross sectional study reported that foods such as chocolate, milk, roasted peanuts or cola have no influence on acne vulgaris conditions [7].

Recently, there has been an increasing number of studies investigating the role of diet as one of the underlying causes of acne vulgaris. Several studies on the effects of ingesting certain dairy products [8-10], carbohydrates, glycemic index (GI) and high glycemic load (GL) diet [11-13] in exacerbating acne vulgaris have been carried out to support the hypothesis that what is eaten may affect the skin. However, the findings of these studies are inconsistent. Retrospective and prospective epidemiological studies reported by Adebamowo et al. [8-10] in the United States of America are the first to provide direct clinical evidence on the association between milk/dairy consumption and acne.

Knowledge on how diet and acne vulgaris is related enables the identification and management of the condition and community education in preventing and improving the acne condition, besides the primary systemic and topical treatment. Therefore, this study was conducted to determine the relationship between dietary variables and acne vulgaris among young adults. Based on previous studies, we hypothesized that high glycemic load diet, milk and dairy products intake, Body Mass Index (BMI) as well as body fat percentage may be the risk factors of acne vulgaris.

## Methods

### Study design

This study was designed as a case–control study. Based on the formula [14], it was calculated that 35 subjects were required to provide 80% power at a significant level of 5%. Standard deviation and magnitude difference was obtained from a previous study on acne patients [11]. After an estimation of 20% drop outs, 44 acne vulgaris patients were recruited as participants in the case group and 44 participants as the control group. Ethical approval was obtained from the Universiti Kebangsaan Malaysia Research Ethics Committee and permission to conduct this study was granted by the Director of Hospital Kuala Lumpur. All participants were given an information sheet about the study and written informed consent was obtained from each respondent.

### Study population

From October 2010 to January 2011, 44 people attending a tertiary hospital Dermatology Clinic in Kuala Lumpur for acne vulgaris treatment were enrolled as participants in the case group. The 44 controls were healthy individuals without acne vulgaris recruited among students and staff members of Universiti Kebangsaan Malaysia Kuala Lumpur Campus. Both the case and control groups were recruited through a convenience sampling method, and then matched by age, gender and ethnicity. People with acne vulgaris aged between 18 to 30 years and referred to dermatologists were included in this study. Patients with chronic diseases such as Systemic Lupus Erythematosus (SLE), diabetic mellitus and heart disease were excluded. Control group participants scored 0 (clear) or 1 (almost clear) for acne severity on the Comprehensive acne severity scale (CASS) [15] as assessed by a qualified dermatologist.

### Measurements

Anthropometric measurements that included body weight, height and body fat percentage were conducted. Body weight was measured using a TANITA digital scale HD-306 (TANITA Corporation, Japan) and height using a SECA 208 body meter (SECA, German) to the nearest 0.1 kg and 0.1 cm respectively. During body weight measurement, participants were required to wear minimal clothing and stand on the centre of the scales with the weight distributed evenly on both feet. Height measurement was performed using the stretch stature method. Participants were asked to stand with the feet together and the heels, buttocks and upper part of the back touching the scale and the head positioned in the Frankfort’s plane. BMI was calculated by dividing the weight (kg) over the square of height (m) and classified according to WHO 2004 classification [16]. Body fat percentage was measured using an Omron Karada Scan Fat Body Analyzer Scale HBF-356 (Omron, Japan).

### Questionnaires

Data related to respondent’s family history, perceptions and beliefs on food that affect acne vulgaris occurrence were obtained using a validated questionnaire with Cronbach-α value of 0.684. Meanwhile, the frequency of milk and dairy products intake were collected through face-to-face interviews using an adapted structured, validated questionnaire [17]. Dietary pattern was assessed using a three day food diary. Participants were asked to record their food intake on two weekdays and one day on a weekend. The three day food diary was attached together with a stamped envelope addressed to the researcher and participants were requested to return the diet record within a period of two weeks. Participants were contacted through phone calls and emails to clarify the information.

### Diet analysis

Daily dietary GL were calculated from the three day food diaries as ∑(GI for food item x its carbohydrate content in grams (g)/100). The GI values were taken from the International Table of Glycemic Index and the Glycemic Load Values [18], International Table of Glycemic Index and the Glycemic Load Values: 2008 [19] and Table of Glycemic Index Value of Selected Malaysian Foods [20]. The GI was estimated by using similar food of known value, if the GI of a food type from Malaysia was not available. Nutrient intakes were calculated using Nutritionist ProTM 2003 software. The Malaysian Food Composition Table (FCT) [21] was used as the nutrient database. However, since the Malaysian FCT did not contain values for vitamin E and selenium, the United States Department of Agriculture (USDA) A National Nutrient Database for Standard [22] and the Food and Nutrient Database for Dietary Studies were used to estimate the intake of these nutrients.

### Statistical analysis

Statistical analyses were conducted using the Statistical Package for the Social Sciences (SPSS) version 19.0. Shapiro-Wilk test was used to test data normality. Descriptive analysis was done in order to elicit the percentage, mean and standard deviation (SD) for quantitative data. Chi-Square test was used to compare milk and dairy products intake between the two groups. Continuous quantitative variables such as anthropometric measurements, glycemic load as well as dietary intake were compared by using unpaired *t*-test. Binary Logistic Regression was used to calculate adjusted odd ratio with the inclusion of confounding factors such as family history, frequency of milk and the intake of dairy products.

## Results

### Demographic data

A total of 94 subjects initially agreed to participate in this study; however 6 subjects were excluded as they did not return their three day diet records. Therefore, 88 subjects, comprising 44 cases and 44 controls aged 18 to 30 years were included in the final analysis of this study. The case group consisted of 29 females (69.5%) and 15 males (34.1%) (Table 1). The subjects included Malays (79.5%) and non-Malays (20.5%) for both groups. Both the case and control groups were mostly single or divorced (86.4%). Significantly more subjects in the control group (95.5%) obtained their education at the tertiary level compared to the case group (68.2%) (p < 0.05). In the case group, 56.8% were employed while 43.2% of them were students. In the control group, the majority (63.6%) were students, and the rest were employed (36.4%).

**Table 1:** Sociodemographic profile and family history of case and control groups

**Parameter**	**Cases (n = 44)**		**Controls (n = 44)**		**P value**
	**n**	**(%)**	**n**	**(%)**	
Gender					
Male	15	(34.1)	15	(34.1)	1.000
Female	29	(65.9)	29	(65.9)	
Ethnicity					
Malay	35	(79.5)	35	(79.5)	1.000
Non Malay	9	(20.5)	9	(20.5)	
Marital status					
Single/Divorced	38	(86.4)	38	(86.4)	1.000
Married	6	(13.6)	6	(13.6)	
Educational level					
Upper Secondary School	14	(31.8)	2	(4.5)	0.002**
Pre-University/Higher education institute	30	(68.2)	42	(95.5)	
Occupation					
Students	19	(43.2)	28	(63.6)	0.054
Employed	25	(56.8)	16	(36.4)	
Income status (monthly), RM (USD)					
≤ RM 2000 (USD6666)	37	(84.1)	40	(90.9)	0.334
> RM 2000 (USD6666)	7	(15.9)	4	(9.1)	
Family history of acne vulgaris					
Yes	36	(81.8)	15	(34.1)	<0.001***
No	8	(18.2)	29	(65.9)	

Chi-Square test.

### Family history of acne vulgaris

More case group participants were found to have a family history of acne vulgaris compared to controls, *χ*2 (1, N = 88) = 20.566 (p < 0.001). Among the cases, 81.8% reported that they had a close relative, such as parents or siblings with acne vulgaris whereas the majority of control group participants had no family history of the disease (Table 1).

### Anthropometric measurements

No statistically significant difference (p > 0.05) was found between the case and control groups in body weight, height and BMI for both sexes with the exception of body fat percentage in male participants (Table 2). Male in the case group had a higher body fat percentage (18.2 ± 5.0%) compared to the males in the control group (16.6 ± 5.6%) (p < 0.05).

**Table 2:** Comparison of mean (±SD) of body weight, height, BMI and body fat percentage between case and control groups according to gender

	**Male (n = 30)**			**Female (n = 58)**		
	**Cases (n = 15)**	**Controls (n = 15)**	**p value**	**Cases (n = 29)**	**Controls (n = 29)**	**p value**
	**Mean ± SD**	**Mean ± SD**		**Mean ± SD**	**Mean ± SD**	
Body weight (kg)	61.7 ± 13.2	64.4 ± 10.0	0.15	61.7 ± 18.3	55.9 ±11.1	0.53
Height (cm)	169.0 ± 8.1	173.9 ± 6.5	0.48	157.9 ± 5.4	156.9 ± 5.0	0.08
BMI(kg/m^2^)	21.8 ± 4.5	21.3 ± 3.1	0.19	24.8 ± 7.2	22.7 ± 4.1	0.72
Body fat (%)	18.2 ± 5.0*	16.6 ± 5.6	0.03	31.2 ± 6.9	29.6 ± 4.7	0.42

* Significant difference using Independent *t*-test (p < 0.05).

### Glycemic load and dietary intake

Overall, the case group had a higher glycemic load (175 ± 35) than the controls (122 ± 28) (p < 0.001) (Table 3). Based on multivariate analysis and taking into account BMI and gender, glycemic load of diet was significantly associated with acne vulgaris occurrence (F(1,86) = 59.412, p < 0.001).

**Table 3:** Dietary glycemic load comparisons between case and control groups

	**Case (n = 44)**	**Control (n = 44)**
	**Mean** ± **SD**	**Mean** ± **SD**
Glycemic load (GL)	175 ± 35***	122 ± 28

***Significant difference using Independent *t*-test, (p < 0.001).

Female acne vulgaris patients had a higher mean of daily energy intake (1812 ± 331) compared to their counterparts in the control group (1590 ± 148 kcal) (p < 0.05) (Table 4). However, no significant differences were found in other nutrients intake between the case and control groups (p > 0.05).

**Table 4:** Gender based comparison of mean (±SD) of energy intake, percentage of macronutrients contribution to energy and micronutrients intake between case and control groups

**Energy and nutrients intake**	**Case (n = 44)**		**Control (n = 44)**	
	**Male (n = 15)**	**Female (n = 29)**	**Male (n = 15)**	**Female (n = 29)**
	**Mean ± SD**	**Mean ± SD**	**Mean ± SD**	**Mean ± SD**
Energy (Kcal)	2056 ± 210	1812 ± 331 *	2011 ± 224	1590 ± 148
Percentage of macronutrients contribution to energy Carbohydrate (%)	50.5 ± 3.6	51.8 ± 6.6	51.7 ± 4.7	53.1 ± 4.3
Fat (%)	33.3 ± 4.1	33.3 ± 5.5	33.7 ± 4.0	31.9 ± 3.9
Protein (%)	16.2 ± 3.5	14.9 ± 3.0	15.1 ± 2.2	15.0 ± 1.7
Micronutrients Vitamin A (RE)	1259.7 ± 432.4	1514.6 ± 503.4	1462.4 ± 515.8	1398.3 ± 470.1
Vitamin E (mg)	5.9 ± 2.6	5.7 ± 3.5	5.6 ± 2.3	4.1 ± 1.9
Fiber (g)	7.6 ± 4.0	6.2 ± 3.2	7.4 ± 3.9	5.3 ± 1.8
Zinc (mg)	6.7 ± 2.3	4.7 ± 2.2	6.3 ± 3.2	4.8 ± 1.8
Selenium (μg)	49.6 ± 34.8	30.7 ± 12.7	27.7 ± 9.9	35.8 ± 17.2

*Significant difference using Independent *t*-test (p < 0.05).

There were no significant differences between case and control participants in energy intake, carbohydrates, fat, protein, vitamin A, vitamin E, fiber, zinc and selenium at the 25th, 50th and 75th percentile (Table 5). Conversely, there was a significant difference in glycemic load at the 50th and 75th percentiles between cases and controls (p < 0.05). At the 75th percentile, glycemic load value of more than 175 increased the risk of occurrence of acne vulgaris by 21 fold. In addition, there was an increase of OR with an increase in the percentile of the dietary glycemic load (25th percentile, OR = 0.05; 50th percentile, OR = 15.75; 75th percentile, OR = 21.00). This showed that the higher the glycemic load, the higher was the risk of acne vulgaris occurrence.

**Table 5:** Glycemic loads of diet, macronutrients and micronutrients intake in percentiles and crude ORs

**Parameter**	**Percentiles**					
	**25**^**th**^	**OR (95% CI)**	**50**^**th**^	**OR (95% CI)**	**75th**	**OR (95% CI)**
Glycemic load	120	0.05 (0.007 – 0.308)	145	15.75** (1.773 – 139.935)	175	21.00** (2.390 – 184.515)
Energy (kcal)	1497	4.08 (1.161 – 13.904)	1782	6.43 (1.662 – 24.860)	2100	1.15 (0.319 – 4.167)
Carbohydrate (g)	197.5	4.67 (1.299 – 16.761)	226.9	8.53 (2.159 – 33.727)	271.3	1.24 (0.340 – 4.558)
Fat (g)	53.1	12.14 (2.655 – 55.537)	62.3	6.80 (1.537 – 30.077)	80.9	8.19 (1.839 – 36.424)
Protein (g)	56.7	4.08 (1.108 – 15.020)	65.0	11.56 (2.822 – 47.356)	83.7	2.83 (0.770 – 10.430)
Vitamin A (RE)	943.4	3.852 (1.086 – 13.661)	1246.7	0.93 (0.282 – 3.062)	1762.2	1.31 (0.393 – 4.388)
Vitamin E (mg)	3.1	4.06 (1.115 – 14.804)	4.5	6.50 (1.603 – 26.360)	6.3	1.35 (0.360 – 5.036)
Fiber (g)	3.6	2.53 (0.750 – 8.522)	5.0	2.14 (0.621 – 7.370)	8.0	1.75 (0.537 – 5.701)
Zinc (mg)	3.6	2.528 (0.750 – 8.522)	4.8	2.528 (0.750 – 8.522)	6.5	1.46 (0.436 – 4.880)
Selenium (μg)	21.1	1.733 (0.525 – 5.723)	28.4	0.45 (0.128 – 1.585)	45.0	2.57 (0.753 – 8.784)

** Significant difference, p < 0.01.

However, after adjusting for confounding variables including family history of acne vulgaris as well as frequency of milk and ice cream consumption using the binary logistic regression analysis, we found that the risk of acne vulgaris remains statistically significant only for the glycemic load value of ≥ 175 with increased odds of having acne vulgaris 25 times higher (Table 6).

**Table 6:** Binary logistic regression model for glycemic load

**Parameter**	**Crude OR**	**(95% CI)**	**Adjusted OR†**	**(95% CI)**
Glycemic load				
<145/≥145	15.75**	(1.773 – 139.935)	1.94	(0.460 – 8.180)
≥175/<175	21.00**	(2.390 – 184.515)	24.96**	(2.285– 272.722)

^†^Adjusted OR for family history, level of education, frequencies of milk and ice cream consumption.

** Significant difference, p < 0.01.

### Milk and dairy products, chocolates and nuts intake

Higher frequency of milk and ice cream intake were positively associated with acne vulgaris occurrence (Table 7). Consumption of milk ≥ once a week increased the risk of acne vulgaris occurrence by 4 times (OR = 3.99, 95% CI = 1.39 - 11.43). Consumption of ice cream ≥ once a week also increased the risk of having acne by 4 times compared to those who did not take ice cream (OR = 4.47, 95% CI = 2.44 - 19.72). The majority of cases (86.4%) drank milk more frequently (≥once a week) compared to 61.4% of the control subjects (p < 0.01). In addition, more cases (56.8%) also consumed a higher frequency of ice cream, (≥once a week) than their counterparts in the control group (22.7%) (p < 0.01). However, no significant differences were found in terms of frequencies of yoghurt, cheese, chocolate and nuts intake (p > 0.05).

**Table 7:** Comparison of milk and dairy products, chocolate and nuts intake frequencies between cases and controls

**Milk and dairy products**	**Frequencies**	**Cases(n = 44)**	**Controls(n = 44)**	**p value**	**Odds Ratio (OR)**	**95% Confidence Interval**
		**n (%)**	**n (%)**			
Milk	≥Once a week	38** (86.4)	27 (61.4)	0.008	3.988	(1.391 - 11.434)
	0 - < Once a week	6 (13.6)	17 (38.6)			
Yoghurt	≥Once a week	10 (22.7)	8 (18.2)	0.597	1.324	(0.467 – 3.749)
	0 - < Once a week	34 (77.3)	36 (81.8)			
Cheese	≥Once a week	10 (22.7)	4 (9.1)	0.080	2.941	(0.846 – 10.229)
	0 - < Once a week	34 (77.3)	40 (90.9)			
Ice cream	≥Once a week	25** (56.8)	10 (22.7)	0.001	4.474	(1.777 – 11.266)
	0 - < Once a week	19 (43.2)	34 (77.3)			
Chocolate	≥Once a week	9 (20.5)	6 (13.6)	0.395	1.629	(0.526 – 5.044)
	0 - < Once a week	35 (79.5)	38 (86.4)			
Nuts	≥ Once a week	9 (20.5)	5 (11.4)	0.244	2.066	(0.613 – 6.558)
	0- < Once a week	35 (79.5)	39 (88.6)			

** Significant difference using Chi-Square test, p < 0.01.

## Discussion

This study suggested that glycemic load has a significant positive relationship with acne vulgaris occurrence after considering factors like BMI and gender. The risk of acne vulgaris decreased with the decreasing percentile of glycemic load, with a significant association noted at the 50th and 75th percentile. The association remained significant after adjustment for other confounding factors. Among the western population, high glycemic load diet was reported as a significant contributor to high acne vulgaris prevalence [23]. The result of this study provides further support to a randomized controlled trial study among male acne patients aged between 15 – 25 years which demonstrated that low glycemic load diet was effective in improving acne vulgaris symptoms [12]. Another study found improvements in biochemical parameters associated with acne vulgaris due to a high-protein, low glycemic-load diet intervention [24]. A study also reported that a low glycemic load diet known as the South Beach Diet was effective to ameliorate the acne conditions of 86.7% of 2995 acne vulgaris respondents in a three months period and reduced the use of conventional systemic as well as topical treatments [25]. In contrast to our study, these studies [12,24,25] found that the effect of the low glycemic load diet was lost when the data was statistically adjusted for changes in BMI.

The underlying mechanism of dietary effect on acne vulgaris formation might be the role of insulin-like growth factor-1 (IGF-1) in facilitating cell proliferation involved in acne vulgaris pathogenesis [26]. Acute hyperinsulinemia due to consumption of high glycemic load diet would cause an increase in IGF-1/insulin-like growth factor binding protein-3 (IGFBP-3) ratio, thus enhancing the effects of IGF-1 [23]. Hyperinsulinemia resulting from high glycemic load diet would also increase circulating androgens and decrease sex hormone binding protein, leading to increased sebum synthesis, which was crucial in acne development.

This study found that frequency of milk and ice cream intake was positively associated with acne vulgaris occurrence. The result is in accordance to a cross-sectional study in South Korea which reported that milk and dairy products intake was associated with acne vulgaris development [27]. They reported that the intake of milk and dairy products was higher among acne vulgaris subjects compared to non-acne subjects [27]. Our data also confirmed epidemiological studies performed in the United States [8-10]. Women, who consumed two or more servings of skimmed milk everyday, were 22% more likely to suffer from severe acne and 44% more likely to develop cystic or nodular acne than those who consumed only one glass of skimmed milk a day [8]. Endocrine factors involved in acne may be affected by milk consumption because milk is an insulinotropic nutrient and has a high insulinemic index [28] which would increase serum insulin and IGF-1 levels [29-32]. Milk produced persistently by pregnant cows contains substantial amounts of steroids and androgen-precursors, which have been suggested to play another role in acne pathogenesis [33,34]. Moreover, another group has proposed a hypothesis for the diet-induced impact of insulin/IGF-1 signaling in acne, as both high glycemic load and dairy proteins increase the serum levels of insulin and IGF-1, important promoters of sebaceous glands and sebaceous lipogenesis [35,36]. In contrast to our study, a positive association between acne with cheese intake was found [8]. The difference may be partly attributed to lower frequency of cheese intake among subjects in this study.

This study confirmed earlier findings that acne vulgaris patients were more likely to have family history of acne vulgaris compared to the control group. In Jordan [37], it was reported that 69.3% of acne vulgaris patients had family history of acne vulgaris while in the United Kingdom, a study involving 458 pairs of monozygotic and 1099 dizygotic female twins [38] found that genetic factors attributed to 81% of acne vulgaris variant and only 19% involved environmental factors. A higher prevalence of acne vulgaris was also found among students with parents having a history of acne vulgaris [39].

Our study has found that acne vulgaris was neither related to BMI nor body fat percentage. This provides further support to a study which discovered no correlation between BMI and leptin levels secreted by adipose tissue with the presence of acne vulgaris or its degree of severity [40]. A retrospective cohort in Turkey [10] also found no significant difference in serum leptin levels between cases and controls. Other studies among female twins [38] and female acne patients [41,42] also found no significant correlation between BMI and acne vulgaris severity among female acne vulgaris patients.

Our study found that yoghurt consumption was not correlated with acne vulgaris occurrence and is consistent with the findings by several studies [8-10]. When added to milk during fermentation process, probiotic bacteria (specifically Lactobacilli) utilize IGF-1 and lowered IGF-1 level in fermented milk by four-fold compared to skim milk [43]. It was hypothesized that increased intestinal permeability occurred in acne vulgaris patients, thus enhancing IGF-1 absorption in the gut [44]. Hence, milk consumption would cause an increased IGF-1 absorption rather than fermented milk products, which have lower levels of IGF-1. This might explain the association of milk with acne vulgaris occurrence in contrast to fermented dairy products such as yoghurt. However, it is proposed that the most important mechanism of milk signalling is the postprandial fast upregulation of insulin secretion and the long-lasting increase in serum IGF-1 levels [35,36]. Recently, lactoferrin-enriched fermented milk was discovered to be effective in decreasing triglycerides in skin surface lipids, resulting in improvement of acne vulgaris symptoms through reduction in sebum production [45].

In contrast to common food beliefs, the present study found no statistically significant association between chocolate and nuts with acne vulgaris occurrences. Chocolate [25] and nuts [23] are commonly believed to cause or aggravate acne condition but previous studies regarding the effects of chocolate and nuts on acne vulgaris condition were inconsistent. An experiment on the effects of milk chocolate bars consumption in subjects with acne vulgaris found no exacerbation in their condition [46]. Subsequently, it was reported that acne lesion counts as well as composition and synthesis of sebum were also not affected after consumption of chocolates bar containing 10 times higher than normal cocoa solids [4]. Another case series study also concluded that chocolate and roasted nuts did not aggravate acne vulgaris condition [7]. Contrary to our findings, a cross sectional study among Koreans involving 783 acne vulgaris patients and 502 controls showed that consumption of nuts among cases were significantly higher than acne-free subjects [27].Thus, there is a clear need to conduct a randomized clinical trial to have a better understanding of the association between these food items with acne occurrence.

A few limitations of the study should be mentioned. Firstly, due to the nature of the study design used, this study was only able to determine the association, but not the cause and effect of diet on acne vulgaris. The severity of acne vulgaris condition should also be taken into account through acne lesion counts on patients in order to relate specific types of food and the frequency of their consumption. Furthermore, a retrospective food frequency questionnaire would be the most appropriate tool to determine the association between dietary intake such as dairy products and acne development. Repeated 3 day food records would have been a better method to calculate glycemic load and determine its association with acne. However, a 3 day food record was used in this study to measure the glycemic load due to the limited study duration. Additionally, this study did not measure the amount of consumed dairy protein, which may be the most important determinant for the acne-promoting effects of milk. Other confounding factors such as stress, inadequate sleep, smoking, alcohol consumption and facial hygiene care should also been taken into account in future studies. Despite these limitations, the findings from our study have provided further support to the existing evidence for the role of diet in acne vulgaris occurrence.

## Conclusions

In conclusion, dietary factors particularly high glycemic load diets as well as higher frequency of milk and ice cream intake were positively associated with acne vulgaris development. Findings from this study further support the hypothesis that dietary factors play a fundamental role in acne vulgaris occurrences. Randomized clinical trials are needed to confirm the role of food items such as peanuts, chocolate and dietary fat in acne occurrence.

## Competing interest

The authors declare that they have no competing interests.

## Authors’ contributions

NHI managed the literature searches, data collection and statistical analysis, and wrote the first draft of the manuscript. ZAM designed the study, developed the protocol, undertook the data interpretation and improved the manuscript. NZA was involved in assessing acne severity, development and improvement of the protocol, and improvement of the manuscript. All authors contributed to and have approved the final manuscript.

## Pre-publication history

The pre-publication history for this paper can be accessed here:

http://www.biomedcentral.com/1471-5945/12/13/prepub
